# 

**Published:** 1972-10

**Authors:** 


The
Bristol Medico-Chirurgical Journal
(Incorporating the Medical Journal of the South-West)
Established 1883

Vol. 87 (iv) OCTOBER 1972 No. 324

### BRISTOL MEDICO-CHIRURGICAL JOURNAL

Editor: N. J. Brown, M.B., F.R.C.P., F.R.C.Path.

Published Quarterly

Annual Subscription £2 Post Free

### BRISTOL MEDICO-CHIRURGICAL SOCIETY

**President 1971-72 : Dr. C. C. Morgans**

**President-elect : Dr. James Macrae.**

**Honorary Secretary : Dr. I. S. Bailey, 7 Percival Road, Bristol 8.**

**Honorary Treasurer : Dr. Frank Ross, Bristol Royal Infirmary, Bristol 2.**

The Society was founded in 1874. In 188 publication of the Journal began and has continued quarterly ever since then. During the years 1949 to 1962 it appeared under the title of "The Medical Journal of the South West".

### THE BRISTOL MEDICO-CHIRURGICAL JOURNAL

### Editorial Committee

Editor Dr. N. J. Brown, Southmead Hospital, Bristol

Members Dr. Rhys Davies.
Dr. M. B. Lennard.
Professor R. C. Wofinden.
Dr. D. W. Wright.
The Hon. Treasurer and Hon. Secretary of the Society.

Business Manager Dr. P. J. F. Baskett, Frenchay Hospital, Bristol.

Secretary Mr. B. P. Jones, The Library, Medical School, University Walk, Bristol BS8 1TD.

### NOTICE TO CONTRIBUTORS

The Journal is especially concerned to provide a place for the recording of medical thought, interests, and practice in and around Bristol. It therefore welcomes articles that, as well as being of immediate interest to members, will document the local and contemporary medical scene for our successors.

Original articles are invited provided that they have not been published elsewhere. References in the text should be by author's name, with year of publication in parenthesis; they should be listed at the end in alphabetical order, the name of each journal or book being given in full—not abbreviated—and the title of the article added. Illustrations should be supplied ready for reproduction. Line drawing should be in Indian ink on firm white paper or board. The author will be informed if it is found necessary to ask him to pay for the making of blocks.

The following contributions will be especially welcome; notes of forthcoming events, meetings held, degrees conferred, staff appointments, honours and public appointments and other local news.

### NEWS AND NOTES

**F.R.C.P. (London)**

K. R. Gough
R. R. M. Harman
M. Hartog
J. H. Middlemiss
P. J. Randle
T. J. Rendle-Short
R. C. Wofinden

**Faculty of Community Medicine of the Royal College of Physicians**

Founder Fellow—R. C. Wofinden

Founder Members—A. W. Macara
R. E. Midwinter
P. N. Dixon
Elizabeth White

### EXAMINATION RESULTS AND DISSERTATIONS APPROVED

### UNIVERSITY OF BRISTOL

### June 1972

**Ch.M.**

Robert Jeffrey CLARKE

**Ph.D.** (Faculty of Medicine)

Linda Frances GREENWOOD
Malcolm Clive ROBERTS
William Albert WATSON (Submitted work)

### QUALIFYING EXAMINATION FOR THE DEGREES OF M.B., Ch.B. (OLD REGULATIONS)

**Pass**

Melvyn Trevor BROWN
Arnold Nigel CADE
Juliet Clare HOLT
Nicholas Brian Michael STEINER
Yat-Cheong WONG

### FINAL EXAMINATION FOR THE DEGREES OF M.B., Ch.B. (NEW REGULATIONS)

**Pass**

Richard Perry ADLAM
Donald Arthur Paul AINSCOW
Robin Leslie ALLUM
Maryse Marie-Louise ARDUIN
Beatrice Marigold AROS-ATOLAGBE
Olatokunbo Ayoka AWOLOWO
Andrew Justin BALL
Ann Lynette BARTLETT-DAVIES
Timothy Ralph Lowndes BAYLEY
Roger BICKERSTAFFE
Angela Margaret BROOKES
David William Wallace BULLIMORE
Shelagh Patricia BULLIMORE
Hilary Mary CANDLER
Jerzy Marek CEMBALA
Arthur Benjamin CHARNAUD
Nasim CHOUDRY
John Martin CLARK
Julian David COLLEDGE
Jacqueline Margery Mary CORNISH
Richard Simon McLeod DAVIS
Naomi Margaret DOE
Liam Joseph DONALDSON
Andrew Frank DOVE
David DREW
Brian DUNKLEY
Paul Nelson DURRINGTON
Michael Russell EVANS
Anthony Dale FALCONER
David John Edmund FARRAND
Christina Mary FEY
Peter John FLEMING
Gerald Martin FRASER
Noel Peter FRENCH
Joseph GALIWANGO
Anne Audrey Mary GEORGE
Robert William HEATH
Ian Daryl HEWETT
Christopher David HOLLAND
John Kenneth Curwood HUMPHREYS
Philip Michael HUTCHINS
Mary Jessica INSKIP
Christopher Sidney IRWIN
Adrian George JACOBS
Francis Hemus JAMES
Kenneth William JARRETT
Susan KINSEY
Lam Son KOH
Thomas Anthony LAVIN
Charles Alexander LAWRENCE
David Francis LEVINE
Jeffrey Alan LINDSAY
Tai Kong Robert LUK
Adrian Philip MANNING
Alfred James MARTIN
Michael Robert MASSEY
Alan Henry MEAKIN
Stephen Frederick METCALFE
Andrew Harland MOORE
Simine Chantal Marie Ghislaine NAHAI
Achac Ali NAJARALI
Andrew Richard Francis NORMAN
James Marcus NORMAN
Christopher Stewart NORRIS
Michael James O'DONOVAN
Lisa Charmian Meredydd PARRY
Mehendra Dahabhai PATEL
Charlotte Frances PATERSON
Susan Rowena PERRY
Nicholas John PICKVANCE
Michael Leslie PRICE
Stephen Rhys PRICE
David Humphrey Alexander REDFORD
Timothy David Richard REYNOLDS
Christopher Mordaunt RICHARDS
Barbara Helen RIDGE
David Vivian RUTTER
Jeffrey Gerald SAFIR
Grace Joy SALAKA
Elizabeth Stewart SAUNDERS
Elizabeth Peta SHAW

Christopher Sydney James SHERVEY
Hugh Stuart SILVEY
Robert Morley SMITH
David STRATON
Jane Marion SYMONDS
Iris Elizabeth SYMONS
John Andrew THOMPSON
Michael Roy THOMSON
Sheila Lilian TOWNSEND
Peter VANEZIS
Gerald Eugene WADDINGTON
Richard Barnes WALLACE
Andrew John WEAVER
Michael Harvey WEBSTER
Michael James WHITEHEAD
Janine Brenda WHITNEY
Ann Helena Mary WILLIAMSON
James Harold WILSON
Paul WILSON
Yvone Shelia WREFORD
Katrin WRIGLEY
Sally May WYON

### HONOURS AND DISTINCTIONS LIST

**Pass With Honours**

Angela Margaret BROOKES

Hilary Mary CANDLER

John Martin CLARK

Gerald Martin FRASER

Mary Jessica INSKIP—with Distinction in Medicine

Christopher Mordaunt RICHARDS—with Distinction in Clinical Pathology, and in Public Health

Michael Roy THOMSON—with Distinction in Surgery

**Pass**

Shelagh Patricia BULLIMORE—with Distinction in Child Health
David DREW—with Distinction in Child Health
Adrian George JACOBS—with Distinction in Medicine
Alfred James MARTIN—with Distinction in Child Health

Dr. C. B. S. Wood, Lecturer in Child Health and Senior Registrar at the Bristol Royal Hospital for Sick Children, has been appointed Professor of Child Health at Saint Bartholomew's Hospital Medical College and the London Hospital Medical College, London.

Mr. K. E. F. Hobbs, Senior Lecturer in Surgery and Consultant Surgeon to the United Bristol Hospitals has been appointed Professor of Surgery at the Royal Free Hospital, London.

Mr. J. P. Mitchell, Consultant Urologist to United Bristol Hospitals and Southmead Hospital, Bristol, has been awarded the Jacksonian Prize by the Royal College of Surgeons.

### ROYAL COLLEGE OF PATHOLOGISTS

**M.R.C.Path.**

A. J. Christie

### The Medical Library

In 1890 the Bristol Medico-Chirurgical Society founded a medical library which in 1892 was combined with that of the Bristol Medical School. This became the University of Bristol Medical Library in 1925 and an agreement in that year confirmed the privilege of Members of the Society to use the University Medical Library.

### A SELECTION OF RECENT ADDITIONS TO THE MEDICAL LIBRARY

ALEXANDER, M.: Microbial ecology. (Wiley)
ANSON, B. J. & McVAY, C. B.: Surgical anatomy. Ed 5. 2 vols. (Saunders)
BACH-Y-RITA, P. & COLLINS, C.C. eds.: The control of eye movements. (Acad. Press)
BAGLEY, C.: The social psychology of the child with epilepsy. (Routledge)
BALDWIN, J. A.: The mental hospital in the psychiatric service: a case-register study. (Oxford Univ. Press for Nuffield Provincial Hospitals Trust)
BANATVALA, J. E. ed.: Current problems in clinical virology. (Churchill Livingstone)
BARBEAU, A. & BRUNETTE, J. R. eds.: Progress in neurogenetics. (Excerpta Medica)
BARBEAU, A. & McDOWELL, F. H. eds.: L-Dopa and parkinsonism. (Blackwell)
BARRETT, A. J. & DINGLE, J. T.: Tissue proteinases. (North-Holland)
BEDFORD, M. A.: A colour atlas of ophthalmological diagnosis. (Wolfe)
BELCHER, E. H. & VETTER, H. eds.: Radioisotopes in medical diagnosis. (Butterworth)
BERGMAN, A. B. et al., eds.: Sudden infant death syndrome. (Washington Univ. Press)
BJÖRKMAN, N.: An atlas of placental fine structure. (Baillière/Williams & Wilkins). Presented by the Association of Applied Biologists.
BOARDMAN, N. K. et al., eds.: Autonomy and biogenesis of mitochondria and chloroplasts. (North-Holland)
BODEMER, C. W.: Modern embryology. (Holt)
BOOTH, C. C. & DOWLING, R. H. eds:. Coeliac disease. (Livingstone)
BOUCHIER, I. A. D. ed.: Seventh symposium on advanced medicine. (Pitman)
BRITISH MEDICAL JOURNAL: Urinary tract diseases. (B.M.A.)
BROWN, D.: Obstetrics for the family doctor. Ed 2 (Pitman)
CALNE, R. Y.: Clinical organ transplantation. (Blackwell)
CHANCE, B. et al., eds.: Probes of structure and function of macromolecules and membranes. 2 vols. (Acad. Press)
CIBA FOUNDATION: Identification of asthma (Churchill)
COHEN, S. et al., eds.: Cellular interactions in the immune response. (Karger)

DAVIES, D. D. & BALLS, M., eds.: Control mechanisms of growth and differentiation. (Cambridge Univ. Press)

DAVIES, J. B. M.: Preventive medicine, community health and social services. Ed 2. (Baillière)

DEELEY, T. J.: Modern radiotherapy—carcinoma of the bronchus. (Butterworth)

DEELEY, T. J.: Modern radiotherapy—gynaecological cancer. (Butterworth)

FARQUHARSON, E. L.: Textbook of operative surgery. Ed 5. (Livingstone)

FEARNHEAD, R. W. & STACK, M. V.: Tooth enamel II. (Wright). Presented by John Wright & Sons Ltd.

FLEISCHMAJER, R. & BILLINGHAM, R. E., eds.: Epithelial-mesenchymal interactions (Williams & Wilkins)

FOLKOW, B. & NEIL, E.: Circulation. (Oxford Univ. Press)

FOX, M. W.: Integrative development of brain and behavior in the dog. (Chicago Univ. Press). Presented by the Association of Applied Biologists

FRANKLIN, T. J. & SNOW, G. A.: Biochemistry of antimicrobial action. (Acad. Press)

GAZZANICA, M. S.: The bisected brain. (Appleton-Century-Crofts)

GOOD, R. A. & FISHER, D. W., eds.: Immunobiology. (Sinauer Associates). Presented by the Association of Applied Biologists.

HAMERTON, J. L.: Human cytogenetics. 2 vols. (Acad. Press)

HARDMAN, G.: Oral surgery for nurses. (Wright). Presented by John Wright & Sons Ltd.

HEALD, F. P. & HUNG, W.: Adolescent endocrinology. (Appleton-Century-Crofts)

HUISMAN, T. H. J. & SCHROEDER, W. A.: New aspects of the structure, function and synthesis of hemoglobins. (Butterworth)

ILLINGWORTH, R. S.: The treatment of the child at home: a guide for family doctors. (Blackwell)

JIRASEK, J. E.: Development of the genital system and male pseudo-hermaphroditism. (Johns Hopkins Press)

KELLNER, R.: Family ill health: an investigation in general practice. (Thomas)

KILLEY, H. C.: Fractures of the mandible. Ed 2. (Wright). Presented by John Wright & Sons Ltd.

KILLEY, H. C. et al.: An outline of oral surgery. Part 2. (Wright). Presented by John Wright & Sons Ltd.

KILLEY, H. C.: Fractures of the middle third of the facial skeleton. Ed 2 (Wright). Presented by John Wright and Sons Ltd.

KIMBALL, A. P. ed.: Prebiotic and biochemical evolution. (North-Holland)

KRAUS, B. S. & JORDAN, R. E.: Human dentition before birth. (Kimpton)

LING, N. R.: Lymphocyte stimulation. (North-Holland)

LIVER CANCER: Proc. of a working conf. held at the Chester Beatty Res. Inst. (Internat. Agency for Res. in Cancer. Scientific Publns., 1) (H.M.S.O.)

LOETHER, H. J.: Problems of ageing. (Wadsworth/Prentice-Hall)

MACK, A.: Full dentures. (Wright). Presented by John Wright & Sons Ltd.

MACMAHON, B. & PUGH, T. F.: Epidemiology: principles and methods. (Little, Brown/Churchill)

MARATKA, Z. & OTTENJANN, R.: Inflammation in gut. (Karger)

### BRISTOL MEDICO-CHIRURGICAL SOCIETY

The sixth meeting of the session was held at the Medical School on 8th March, 1972. Dr. N. J. Brown showed pathological specimens and Dr. G. R. Airth demonstrated skiagrams. Mr. A. G. McPherson, Consultant Surgeon at Southmead Hospital addressed the Society on "Some Surgical Problems of the Newborn". This paper will be published in a future number of the Journal.

At the seventh meeting on 12th April 1972, Prof. M. A. Epstein, Professor of Pathology in the University of Bristol, spoke on "Some Unusual Problems relating to Suspected Human Tumour Viruses". Dr. N. Gubbay showed pathological specimens and Dr. P. Ashman James demonstrated skiagrams.

The final meeting of the 1971-72 session, on 10th May 1972 was the occasion of an address entitled "Doomsday Tomorrow?" given by Mr. Crispin Gill, Editor of *The Countryman.*

At all the above meetings Mr. B. P. Jones, the medical librarian, arranged his usual display of medical books.

### SOUTH-WEST ORTHOPAEDIC CLUB

A Meeting of the Club was held in Plymouth on the 29th April, 1972.

**SYMPOSIUM ON AMPUTATIONS**

**Amputations of the Upper Limb**—Dr. I. Fletcher (Roehampton).

**Present Problems in Limb Fitting**—Dr. C. E. Halliday (Limb Fitting Centre, Exeter and Plymouth).

**Future Trends in Limb Fitting**—Dr. R. G. Redhead (Roehampton).

**The Waterhouse-Friderichsen Syndrome and its Orthopaedic Consequences** — Surg. Commd. T. Hampton, R.N,. Surg. Capt. P. D. G. Pugh, R.N.

**Venous Gangrene**—Mr. N. Seymour (Plymouth)

**Surgical Treatment of Cervical Spondylosis**—Mr. W. E. Strachan (Plymouth) spoke of the treatment of neurological complications in cervical spondylosis by anterior cervical decompression and fusion. His comments were based upon a pilot study of 52 patients individually assessed in follow-up periods of 12 to 45 months. There were 33 males and 19 females, with a mean age of 56 years and an average duration of symptoms of 3 years. All patients had extensive conservative measures prior to surgery. Thirteen patients had pure radiculopathy, 11 myelopathy and 28 had a mixed picture; all were considerably incapacitated and 12 were either bedridden or barely ambulant. Radiological levels were determined by myelography and 79 levels were operated upon. The operation was the Harris modification of the Cloward technique. There were 26 single levels, 25 double and 1 had three levels operated on. Maximum lateral decompression of the nerve roots and radicular arteries was attempted and fusion was achieved with bone dowels obtained from the iliac crest. Of the patients under 60 years of age, 72% returned to their previous occupation, 16% obtained lighter jobs and 12% were not gainfully employed. The results were not influenced by age, sex or severity of symptoms nor the levels affected or treated. Five cases did not obtain material benefit from surgery and of these 4 had coexistent neurological disorders.

**Patellectomy for Chondromalacia Patellae**—Surg. Lt. Commd. A. C. Chakraverty, R.N., discussed the place of patellectomy in treatment of chondromalacia patellae. Chondromalacia is a particularly common problem in the Royal Navy. The overall results of patellectomy were good but they often tend to be disappointing in the adolescent. Undue prolongation of conservative treatment and multiple operations on the same knee were observed in the majority of the patients obtaining poor results after patellectomy. A moderate to extensive degree of new bone formation in the patellar bed after excision was seen in 54.5% of the patients who had been invalided from the Service after patellectomy. He suggested that further investigation was necessary into the problem of this new bone formation, which usually has an adverse effect on the outcome of the operation. The initial results of patelloplasty were encouraging and this procedure might well prove to be a better operation for chondromalacia, particularly for the younger age group and for females where a good cosmetic result is more desirable.

**The Sliding Nail for Intracapsular Fractures of the Femoral Neck**—Dr. S. K. Mukerjea (Plymouth) argued against the increasing tendency to carry out replacement of the femoral head for intracapsular fractures of the neck of the femur. He had been able to obtain good early results using a sliding nailplate. He stressed the need to obtain a good reduction with the head in slight valgus, and acurate internal fixation was essential.

**An Operation for Dupuytren's Contracture**—Dr. J. G. Hazelwood (Plymouth) described a technique for Dupuytren's Contracture involving two or more fingers. A palmar V incision, one limb extending to the ulnar border of the little finger the other to the web space of the index and middle fingers, is made. The skin flap, and where possible the subcutaneous fibro-fatty layer, is reflected distally. This gives a good exposure allowing a radical fasciectomy, avoiding damage to the neuro-vascular bundles, to be done under direct vision. Contractures of the metacarpal-phalangeal joints can be dealt with but proximal interphalangeal joint contractures may require a separate Z-plasty. Skin closure is obtained by converting the V into a Y plasty. There were no complications in 19 cases except sloughing of the skin flap in one patient who had had two previous operations on the hand.

**Reflections on Six Years' Experience of A.O. Techniques**—Surg. Capt. P. D. G. Pugh, R.N.

**Some Lesions of the Clavicle**—Mr. F. T. Wheeldon (Plymouth) gave an account of 4 patients with unusual problems involving the clavicle. One patient, who had had a fracture dislocation of the sterno-clavicular joint internally fixed with a Kirschner wire, developed an unusual chest complication due to migration of the Kirschner wire down into the chest and into the pericardium.

**The Pelvic Aspect of the Acetabulum in Charnley's Arthroplasty**—Mr. N. Seymour (Plymouth). Following a case of thrombosis of the external iliac vein and a case of spasm of the pelvic colon after Charnley's arthroplasty, the acetabulum was studied. In a number of post mortem dissections the floor of the acetabulum was drilled and it was found that the pelvis was penetrated between the internal and external iliac veins and always very close to the obturator nerve. The anatomy varied so that sometimes either vein was close to the drill point. Also the thickness of the acetabular floor and the bulk of the obturator internus muscle varied considerably. In order to study the temperature reached in the vicinity of tissues deep to the floor of the acetabulum, some Charnley arthroplasties were performed using a posterior approach. The drill hole was palpated from the sciatic notch and a thermocouple placed on the pelvic side of obturator internus in the vicinity of the hole. Measurements have to date been carried out in eight patients, some with and some without the use of the stainless steel restrainer. The rise in temperature after insertion of the cement is small, being a maximum of 3°C without the restrainer and with an average rise of only $\frac{1}{2}$°C when the restrainer is used.

**Nelson, His Wounds and His Ceramics**—Surg. Capt. P. D. G. Pugh, R.N.

### *INDEX TO VOLUME 87*

*AUTHORS*

Bowdler Ingrid, M. .... 53

Brown, C. A. .... 1

Eastham, R. D. .... 5

de Fonseka, C. P. .... 37

Jancar, J. .... 23

Johnson, C. .... 31

Mackenzie, Vivien .... 33

Pengelly, C. D. R. .... 33

Penry, J. B. .... 31

Radford, P. .... 19

Roberts, J. L. .... 37

Walker, W. L. .... 15

*SUBJECTS*

Accidents, in home .... 37
Adolescence, crises in .... 15
Anticoagulent therapy .... 5
Barium meals, service for G.P.'s .... 31
Brentry Hospital .... 23
Bristol Medico-Chirurgical Society ... 13, 22, 59
Clomiphene .... 53
Glaucoma .... 1
Medical library .... 10, 58
Naturalist, psychology of .... 19
News and notes .... 22, 57
Obituaries — Capper W. M. .... 9
— McAneny, T. M. .... 21
Red cell volume .... 33
South West Orthopaedic Club .... 13, 59
West Country Rheumatism Club .... 14

